#  Avian Influenza Virus H5 Strain with North American and Eurasian Lineage Genes in an Antarctic Penguin 

**DOI:** 10.3201/eid2212.161076

**Published:** 2016-12

**Authors:** Gonzalo P. Barriga, Dusan Boric-Bargetto, Marcelo Cortez San Martin, Víctor Neira, Harm van Bakel, Michele Thompsom, Rodrigo Tapia, Daniela Toro-Ascuy, Lucila Moreno, Yesseny Vasquez, Michel Sallaberry, Fernando Torres-Pérez, Daniel González-Acuña, Rafael A. Medina

**Affiliations:** Pontificia Universidad Católica de Chile, Santiago, Chile (G.P. Barriga, R.A. Medina);; Pontificia Universidad Católica de Valparaíso, Valparaíso, Chile (D. Boric-Bargetto, F. Torres-Pérez);; Universidad de Santiago, Santiago (M. Cortez-San Martin, D. Toro-Ascuy, Y. Vasquez);; Universidad de Chile, Santiago (V. Neira, R. Tapia, M. Sallaberry);; Icahn School of Medicine at Mount Sinai, New York, New York, USA (H. van Bakel, R.A. Medina);; Universidad de Concepción, Concepción, Chile (M. Thompsom, L. Moreno, D. González-Acuña);; Millennium Institute on Immunology and Immunotherapy, Santiago (R.A. Medina)

**Keywords:** avian influenza, avian influenza virus, AIV, LPAIV, viruses, influenza, penguin, Antarctica, molecular epidemiology, zoonoses

**To the Editor:** Previous studies have reported avian influenza virus (AIV)–positive serum samples obtained from Adélie (*Pygoscelis adeliae*), chinstrap (*Pygoscelis antarcticus*), and gentoo (*Pygoscelis papua*) penguins (*1**–**4*). Only recently was an H11N2 subtype virus isolated from Adélie penguins in Antarctica (5). We performed AIV surveillance in the Antarctic Peninsula to identify the strains currently circulating in different penguins species in this area. 

During 2015–2016, we sampled penguin colonies from 9 locations on the Antarctic Peninsula. We collected 138 blood samples from Adélie penguins at Ardley Island (62°13′S, 58°56′W), Arctowski Base (62°9′S, 58°28′W), and Bernardo O’Higgins Base (63°19′S, 57°53′W) and identified 5 serum samples positive for influenza. We also collected 513 cloacal swabs from Adélie, chinstrap (Technical Appendix Figure 1, panel A), and gentoo penguins from Mikkelsen Harbor (63°54′S, 60°47′W), Dorian Bay and Port Lockroy (64°48′S, 63°30′W), Pleneau Island (65°06′S, 64°04′W), Brown Base (64°53′S, 62°52′W), Orne Harbor (64°37′S, 62°32′W), and Aitcho Island (62°23′S, 59°46′W) during January–March of 2 consecutive seasons (2015 and 2016; Technical Appendix Figure 1, panel B). Quantitative reverse transcription PCR (RT-PCR) analysis of the matrix segment (*6*) identified 21 positive AIV samples from penguins (8 chinstrap, 13 gentoo) on Aitcho Island, demonstrating the presence of AIV in 2 additional penguin species in a new location in Antarctica.

Using multisegment RT-PCR performed with influenza-specific universal primers, we amplified all 8 virus segments from a chinstrap penguin specimen, which yielded cDNA products suitable for next-generation sequencing with a HiSeq 2500 System (Illumina, San Diego, CA, USA). This virus was subtyped as an H5N5 and named A/chinstrap_penguin/Antarctica/B04/2015 (H5N5). Analysis of its cleavage site confirmed this was a typical low pathogenicity AIV (LPAIV) containing cleavage motif PQRETRGLF (*7*).

To trace the origin of this H5N5 virus, we performed phylogenetic analyses of its hemagglutinin and neuraminidase genes (Figure, panels A, B; Technical Appendix Figures 2, 3). The hemagglutinin gene was placed into a clade within the H5 American LPAIV lineage, clustering with AIVs isolated from ducks in the United States during 2007–2014 and blue-winged teals from Guatemala in 2010 (Technical Appendix Figure 2). This finding suggests a possible introduction of this H5 AIV into Antarctica via the Pacific or the Mississippi–American flyways, although we cannot rule out that this H5 strain is endemic to other South America locations.

The timing of arrival of migratory birds that breed in Antarctica (e.g., skua, shags, petrel, and gulls) overlaps with that of the penguins as they return to colonies for breeding and nesting during the summer in the Southern Hemisphere. These birds share a habitat, enabling close contact (*5*,*8*) and introducing the possibility of AIV spillover from flying birds to penguins. The chinstrap penguin H5 strain also clustered near the H5 strain isolated in 2008 from a kelp gull (*Larus dominicanus*) in Chile (*9*), indicating a potential route of transmission and introduction of AIV into Antarctic penguins (Figure, panel A). Kelp gull colonies are found in the Antarctic, the sub-Antarctic territory, and along the coastline of Chile and Argentina. Hence, gulls and other intermediate vector hosts, such as the south polar skua (*Stercorarius maccormicki*), might represent natural reservoirs that play a role in the introduction and maintenance of AIVs into Antarctica.

**Figure:**
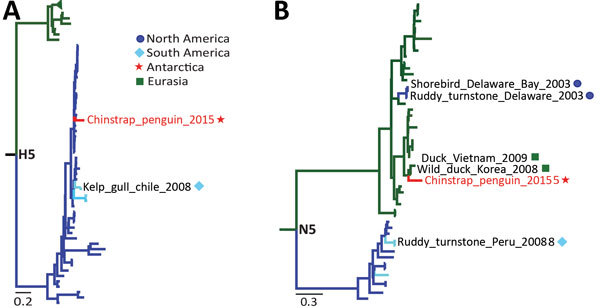
Low pathogenicity avian influenza virus (AIV) (H5N5) found in Antarctic penguin. A) Phylogenetic analysis of the HA gene showing its relationship to H5 low pathogenicity North American lineage viruses. B) Phylogenic analysis of the NA gene showing its relationship to N5 viruses from Eurasia. Antarctic strains: red lines, stars; Eurasian strains: green lines, squares; North American strains: dark blue lines, circles; South American strains: light blue lines, diamonds. Sequences were selected from public databases to cover a wide diversity of AIV strains from different years and geographic locations and aligned with MUSCLE (http://www.drive5.com/muscle/). The maximum-likelihood trees of 325 HA and 319 NA nucleotide sequences were constructed with MEGA6 (http://www.megasoftware.net) and IQ-TREE on the IQ-TREE web server (http://www.cibiv.at/software/iqtree/) by using the maximum-likelihood method with 1,000 ultrafast bootstrap replicates. Summarized trees are shown for the H5 and N5 clusters. Further details are provided in Technical Appendix Figures 2, 3**.** The best-fit model of substitution was found by using the auto function on the IQ-TREE web server and Akaike information criterion. Scale bars indicate nucleotide substitutions per site. AIV, avian influenza virus; HA, hemagglutinin; NA, neuraminidase.

The chinstrap penguin neuraminidase segment clustered within a Eurasian N5 clade that includes sequences from 2001–2010 (Figure, panel B; Technical Appendix Figure 3). The closest sequences were isolated from wild ducks from South Korea in 2008 (GenBank accession no. JX679163) and Vietnam in 2009 (GenBank accession no. AB593481). Eurasian N5s have sporadically been found in ruddy turnstones (*Arenaria interpres*) and an unidentified shore bird at Delaware Bay (GenBank accession nos. CY144466.1, CY144458.1, and CY102738.1). This finding suggests a plausible entryway of this gene into Antarctica from South America through the Atlantic or Pacific-American flyway, which are common routes used by shore birds, such as the ruddy turnstone, white-rumped sandpiper (*Calidris fuscicollis*), and red knot (*Calidris canutus*) (*10*).

As previously suggested for H11N2 viruses from Antarctica, our data supports the idea that these AIVs are evolutionarily distinct from other AIVs (*5*). This H5N5 strain is a contemporary reassortant virus related to North American and Eurasian strains.

The positive animals we identified originated from a single location on the Antarctic Peninsula, which suggests recent introduction of this AIV H5N5 in the colonies sampled. Antarctica is refuge for most penguin colonies, including the near-threatened emperor penguins. Previous reports suggested that AIV could have caused Adélie penguin chick death (*3*). Four positive samples (including the sequenced virus) were obtained from juvenile chinstrap penguins that were weak, depressed, and possibly ill (i.e., they had ruffled feathers, lethargy, and impaired movement). Thus, additional studies are warranted to assess the health and conservation status of resident bird species and potential pathologic effects of AIV.

These data provide novel insights on the ecology of AIV in Antarctica. Our findings also highlight the need for increased surveillance to understand virus diversity on this continent and its potential contribution to the genetic constellation of AIV in the Americas.

Technical Appendix Phylogenetic analyses of hemagglutinin and neuraminidase gene segments of avian influenza virus H5N5 obtained from chinstrap penguin in Antarctica in 2015, showing North American and Eurasian origins.
