# Supplementary material for: Avian Influenza Virus H5 Strain with North American and Eurasian Lineage Genes in an Antarctic Penguin
Source: Emerg Infect Dis. 2016 Dec;22(12):2221–3. doi: 10.3201/eid2212.161076 (PMC5189164; doi:10.3201/eid2212.161076)
Supplement: Technical Appendix — Phylogenetic analyses of hemagglutinin and neuraminidase gene segments of avian influenza virus H5N5 obtained from chinstrap penguin in Antarctica in 2015, showing North American and Eurasian origins. [file 16-1076-Techapp-s1.pdf]

Article DOI: http://dx.doi.org/10.3201/eid2212.161076

Avian Influenza Virus H5 Strain with North American and Eurasian Lineage Genes in an Antarctic Penguin

Technical Appendix

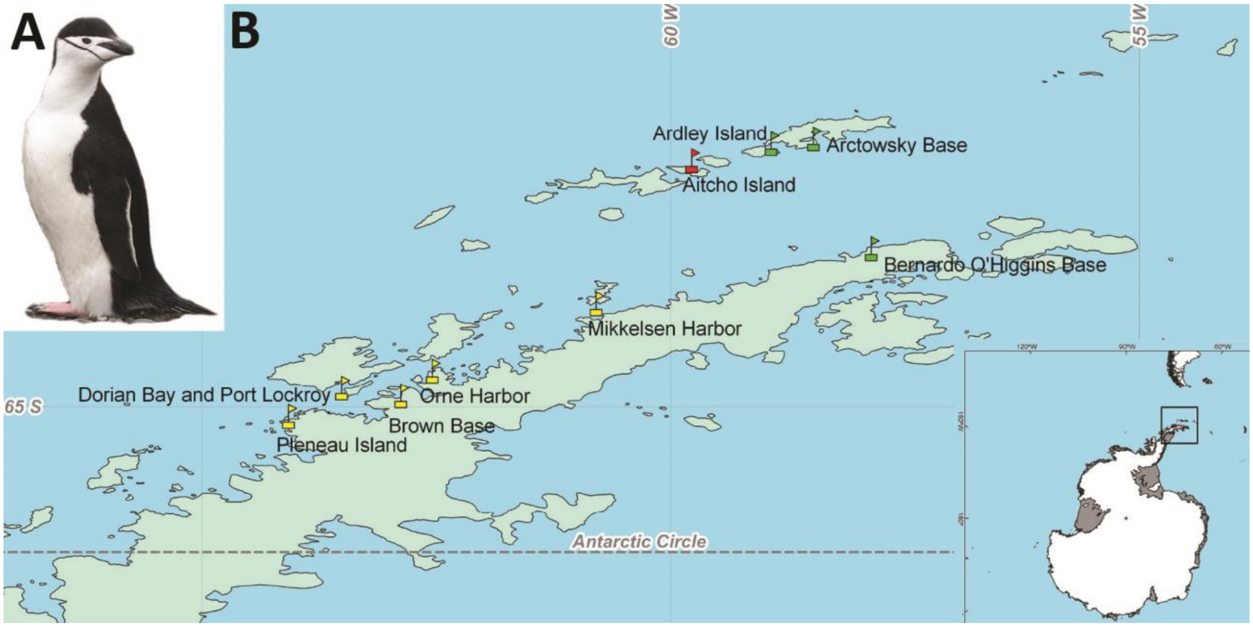

Technical Appendix Figure 1. Low pathogenicity avian influenza virus (AIV) (H5N5) found during penguin sampling of 9 locations on the Antarctica Peninsula. A) Chinstrap penguin, the species from which the novel influenza A (H5N5) virus strain was obtained. B) Antarctic Peninsula. Colored flags indicate sample type: green, serum samples; yellow, cloacal swab samples; red, virus-positive swab samples.

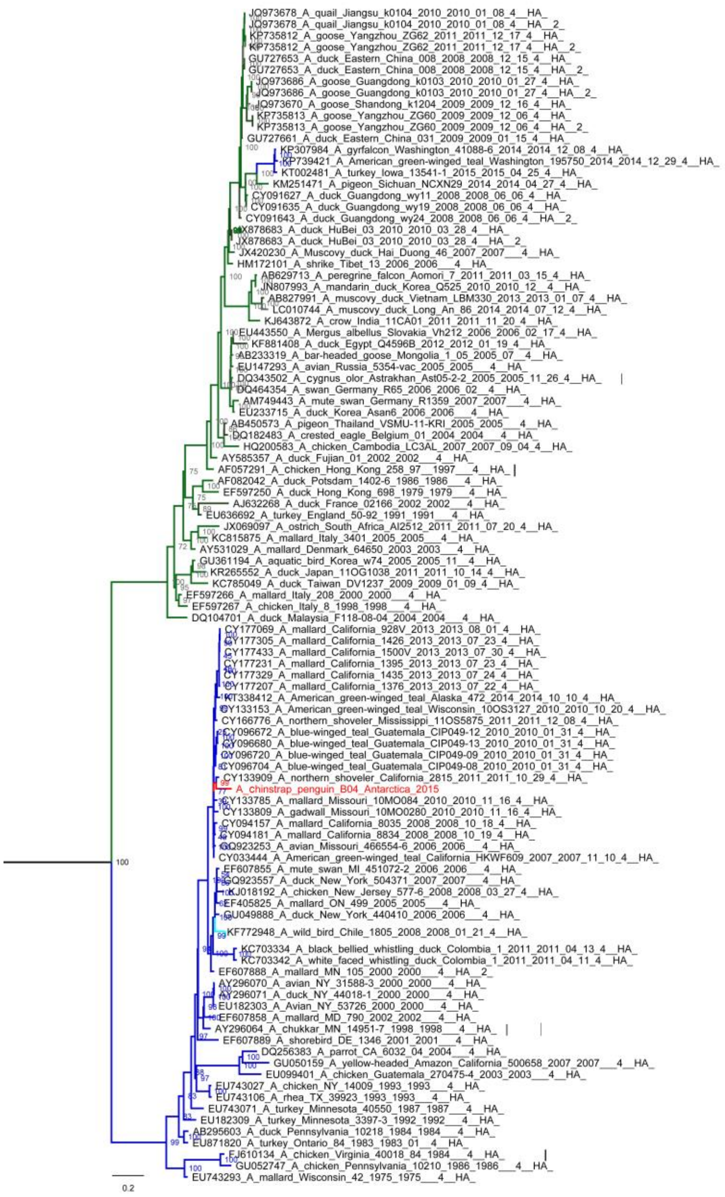

Technical Appendix Figure 2. Phylogenetic analysis of hemagglutinin (HA) gene segment obtained from chinstrap penguin in Antarctica in 2015 reveals a North American origin. HA gene A/chinstrap/Antarctica/B04/2015 (GB:KX458007; in red) clusters within the low pathogenicity North American H5 lineage. Sequences were selected from public databases to cover a wide diversity of AIV strains from different years and geographic locations and aligned with MUSCLE. The maximum-likelihood tree of 325 HA nucleotide sequences was constructed with MEGA6 and IQ-TREE on the IQ-TREE web server (http://www.cibiv.at/software/iqtree/) by using the maximum-likelihood method with 1,000 ultrafast bootstrap replicates. The best-fit model of substitution was found by using the auto function on the IQ-TREE web server and Akaike information criterion. Scale bar indicates nucleotide substitutions per site. AIV, avian influenza virus; HA, hemagglutinin.

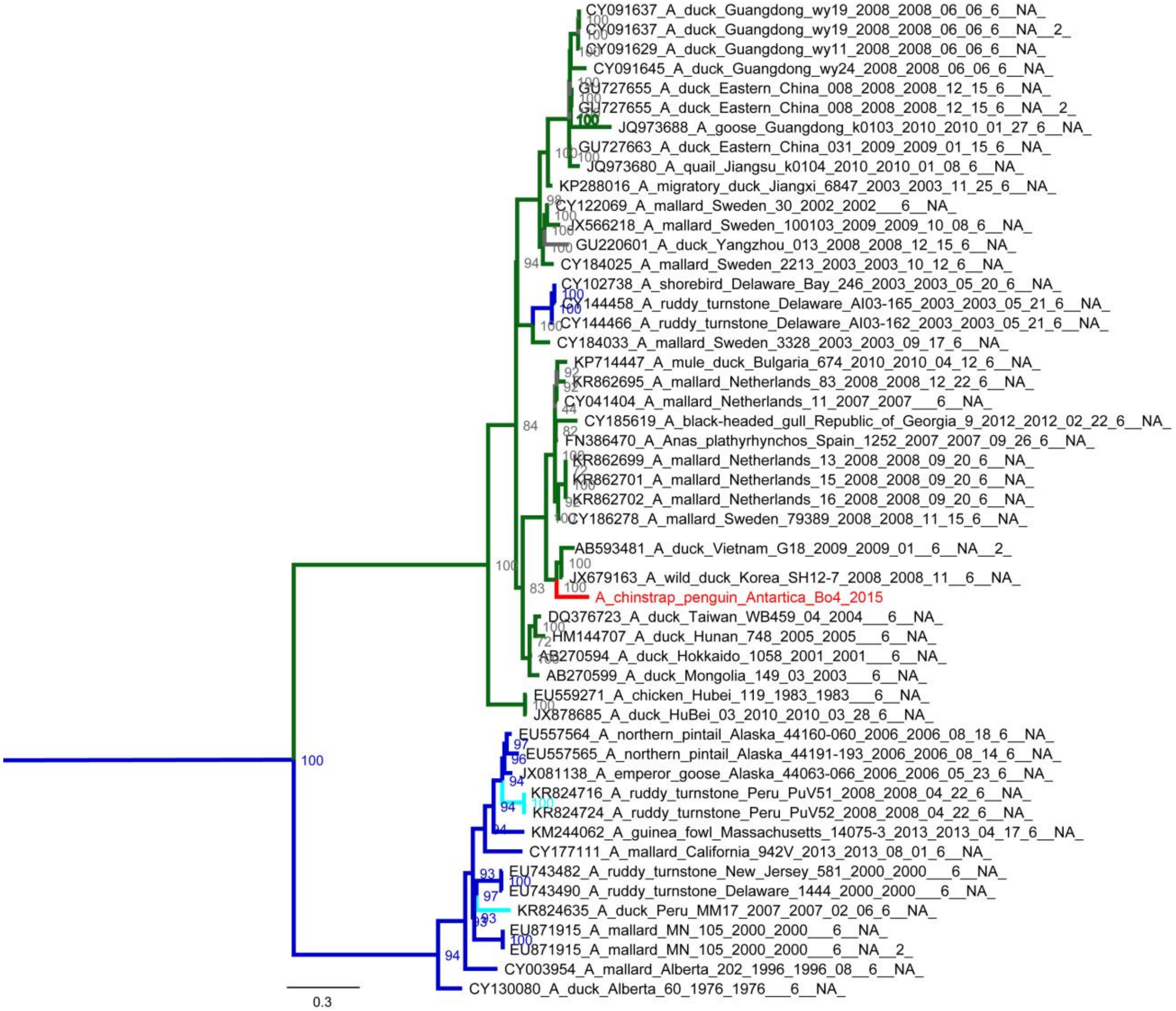

Technical Appendix Figure 3. Phylogenetic analysis of neuraminidase (NA) gene segment obtained from a chinstrap penguin in Antarctica in 2015 reveals a Eurasian origin. NA gene A/chinstrap/Antarctica/B04/2015 (GB:KX458006; in red) clusters within the Eurasian N5 clade. Sequences were selected from public databases to cover a wide diversity of AIV strains from different years and geographic locations and aligned with MUSCLE. The maximum-likelihood tree of 319 NA nucleotide sequences was constructed with MEGA6 and IQ-TREE on the IQ-TREE web server (http://www.cibiv.at/software/iqtree/) by using the maximum likelihood-method with 1,000 ultrafast bootstrap replicates. The best-fit model of substitution was found by using the auto function on the IQ-TREE web server and Akaike information criterion. Scale bar indicates nucleotide substitutions per site. AIV, avian influenza virus; NA, neuraminidase.
